# Heat generated during dental treatments affecting intrapulpal temperature: a review

**DOI:** 10.1007/s00784-023-04951-1

**Published:** 2023-04-06

**Authors:** Xin Er Lau, Xiaoyun Liu, Helene Chua, Wendy Jingwen Wang, Maykon Dias, Joanne Jung Eun Choi

**Affiliations:** Sir John Walsh Research Institute, Faculty of Dentistry, University of Otago, 310 Great King Street, Dunedin, 9016 New Zealand

**Keywords:** Pulp temperature, Dental equipment, Heat generation

## Abstract

**Introduction:**

Heat is generated and transferred to the dentine-pulp complex during various dental procedures, such as from friction during cavity preparations, exothermic reactions during the polymerisation of restorative materials and when polishing restorations. For in vitro studies, detrimental effects are possible when intra-pulpal temperature increases by more than 5.5°C (that is, the intra-pulpal temperature exceeds 42.4°C). This excessive heat transfer results in inflammation and necrosis of the pulp. Despite numerous studies stating the importance of heat transfer and control during dental procedures, there are limited studies that have quantified the significance. Past studies incorporated an experimental setup where a thermocouple is placed inside the pulp of an extracted human tooth and connected to an electronic digital thermometer.

**Methods:**

This review identified the opportunity for future research and develop both the understanding of various influencing factors on heat generation and the different sensor systems to measure the intrapulpal temperature.

**Conclusion:**

Various steps of dental restorative procedures have the potential to generate considerable amounts of heat which can permanently damage the pulp, leading to pulp necrosis, discoloration of the tooth and eventually tooth loss. Thus, measures should be undertaken to limit pulp irritation and injury during procedures. This review highlighted the gap for future research and a need for an experimental setup which can simulate pulp blood flow, temperature, intraoral temperature and intraoral humidity to accurately simulate the intraoral conditions and record temperature changes during various dental procedures.

## Introduction

Human teeth consist of hard components (enamel, dentine, cementum), soft pulp tissue and sensory fibres [1]. Human teeth are regarded as a sensory tissue with the pulp, a soft connective tissue, containing nerve fibres and nerve endings extending into the dentinal tubules. These pulpal nerve terminals are crucial in sensing thermal stimuli [2–4]. Although heat transfer in human teeth is a common occurrence in both daily life and clinical dentistry, there is a lack of knowledge regarding the actual amount of heat transfer that takes place during dental procedures. This is important as trauma must be limited to a stressed pulp, where the accumulation of thermal, microbial, chemical and mechanical can compromise its vitality. Zach and Cohen [5] reported that an increase of 5.5°C in temperature can result in irreversible pulpitis and has since been the threshold cited by subsequent studies as the maximum temperature increase the dental pulp can endure. Although this value may have limited clinical relevance, it provides a value to which the results of other in vitro studies can be compared to. There are various stages during the dental treatment which generate heat, affecting the intrapulpal temperature: from cutting of the tooth structure by high-speed dental handpieces (HSDH), exothermic reactions during the polymerisation of light or self-cured restorative materials and during the polishing step. However, little is known about the effect of various factors which can increase the intrapulpal temperature. Moreover, since measuring the intrapulpal temperature in human subjects is both unethical and unfeasible, previous studies have adopted in vitro simulation models to conduct research on the change of intrapulpal temperature.

This review paper attempts to provide a comprehensive understanding on the heat generation during dental treatments affecting intrapulpal temperatures. To address this, firstly, the human tooth structure and heat transfer mechanism of enamel and dentine will be explained. Secondly, factors affecting the intrapulpal temperature during tooth preparation (cutting), crown fabrication, light curing and polishing will be discussed. Lastly, in vitro and in vivo methodologies used to study the intrapulpal temperature will be discussed, along with its opportunities and challenges. The objective of this review is to give an overview of the current research done on the heat generation during dental procedures and highlight the areas for future research to improve the understanding of the various factors that can affect the intrapulpal temperature.

## Structure of human teeth

Enamel is the highly mineralised outermost layer, which is directly affected during restorative treatment. Below this is dentine, a mineralised connective tissue layer composed of an organic matrix of collagenous proteins [6]. Dentine accounts for most of the tooth structure by both weight and volume. It exhibits a complex hierarchical structure of organic and inorganic components, composed of approximately 70% mineral and 20% organic materials (mainly type I collagen) and 10% water by weight [6, 7]. In essence, dentine serves as the elastic foundation that supports the outermost hard and brittle enamel layer, while also acting as a protective medium for the innermost soft tissue, the pulp [1]. However, perhaps the most distinct feature of this layer’s microstructure is the network of long channels—the dentinal tubules. These extend outwards from the innermost pulp layer towards the exterior cementum or dentine-enamel junction (DEJ) [1, 6–10].

The dental pulp is a highly vascularised tissue encased in hard dentinal walls, containing a large amount of connective tissue, nerve fibres and sensory nerve endings [7]. Its innate ability to heal and repair itself has been previously studied, with the combination of the inflammatory response as well as both the proliferation and differentiation of numerous cell types combining to achieve the repair of the pulp-dentine tissue [11]. Regardless, the pulp is still vulnerable to impairment, particularly to heat exposure during tooth preparation and extensive restorative procedures. Pulp insults are mainly results of heat changes, desiccation, exposure to chemicals and bacterial infection [12]. Normal intrapulpal baseline temperature appears to range between 34 and 35°C [13], with increases in intrapulpal temperature exceeding 42 to 42.5°C sufficient to cause irreversible damage [13, 14]. This is of particular importance as an increase in intrapulpal temperature does not necessarily produce an increase in pulpal blood flow. Consequently, for the pulp which may already be dealing with the effect of thermal changes from tooth preparation, any previous inflammatory changes and limited perfusion may lead to the potential loss of pulpal vitality [15]. The effects of different harmful insults are cumulative, and where possible, dental clinicians must avoid materials and procedures which may contribute to the potential for iatrogenic damage to the pulp [16].

For in vitro studies, irreversible biological effects result when intra-pulpal temperature increases by more than 5.5°C (that is, the intra-pulpal temperature exceeds 42.4°C). It was found that 15% of the experimental teeth developed irreversible pulpitis or necrosis when this temperature was reached [5]. This is shared by another study which determined the temperature range for reversible damage to be between 42 and 42.5°C [17]. Overestimation of the pulp temperature changes in in vitro studies is probable, with the lack of blood and dentine fluid flow, and lack of periodontal tissues [18–20].

### Mechanism of thermal insult to a human tooth

When heat is transferred to the pulp, it can cause various histopathological changes which may lead to irreversible injury. Unlike heat transfer to other materials, the thermal behaviour of teeth is a heat conduction process, combined with its physiological processes, such as dentinal fluid flow and pulpal blood flow [7]. The mechanism of injury includes protoplasm coagulation, expansion of the liquid in the dentinal tubules, increased outwards flow from the tubules, vascular injuries and tissue necrosis [12, 13, 16, 21]. Moreover, because of the variance in thermophysical properties and microstructure between the layers in human teeth, heat transfer may also result in thermal stresses that lead to cracking within the different layers [7, 22].

It is thought that an intrapulpal temperature rise above 43°C activates nerve fibres, leading to a reactive increase of blood circulation which assists in the dissipation of any heat advancing towards the dental pulp [7]. Additionally, the surrounding periodontal tissues could also play a significant role in promoting heat convection, thus limiting the intrapulpal temperature rise [14]. Although the flow of dentine fluid can enhance the heat transfer within the pulp upon heating, it is the pulp microcirculation of blood that plays an important role in the thermoregulation of pulpal soft tissue. In essence, the pulp blood flow rate is practically constant within the range of 33 to 42°C but increases significantly when the temperature rises above 42°C. Perfused blood works as a heat sink under heating and as a source of heat when subjected to cooling. Yet, the overall influence of pulpal blood flow on heat transfer is thought to be minimal due to its relatively low blood volume [7].

In addition, several other biological factors impact on whether the pulp tissue undergoes irreversible effects. This includes the amount of water content in the pulp, the changes in pulp blood and dentinal fluid flows, previous injury to the pulp, the health of the tissues, remaining dentine thickness and insulating quality, duration of insult and the surface area of exposed dentinal tubules [23–28]. Alternative consequences, such as necrosis and alveolar bone loss, and even ankylosis can also occur when intrapulpal temperatures increase by 3 to 10°C during tooth preparation [29, 30]. Higher and longer lasting temperature peaks, and specifically those exceeding the 5.5 °C increase threshold, may lead to pulpal necrosis, and an excessive temperature increase of 3–10 °C can lead to periodontal malformations (e.g. alveolar bone necrosis, bone loss and ankylosis) [29, 30].

### Tooth heat transfer

The relatively low values for thermal conductivity (TC) and diffusivity of enamel and dentine help protect the deeper tissues from thermal insults [31]. Additionally, the characteristic arrangements of its inner structures have a significant influence on heat excursion in teeth [7]. Nevertheless, greater attention is given to dentine since it is often the layer in direct contact with provisional materials and the layer likely to be involved in the heat transfer that takes place from the surface of the tooth preparation to the pulp chamber.

Even though both enamel and dentine are hard components with a high percentage of mineral content, their thermophysical properties are different. TC indicates the ability of a material to conduct heat and the TD is the measure of the speed with which a temperature change will proceed through an object [32]. The TD and TC of enamel are approximately 2.5 and 1.6 times larger than dentine, respectively [33]. Dental pulp is involved in the maintenance of tooth vitality and is vulnerable to heat changes without the protection of the enamel and dentine layers. The TC and TD of enamel and dentine are relatively low compared to those of the pulp; therefore, these two layers are effectively thermal insulators and protect the pulp from deleterious thermal irritation [7].

**Fig. 1:**
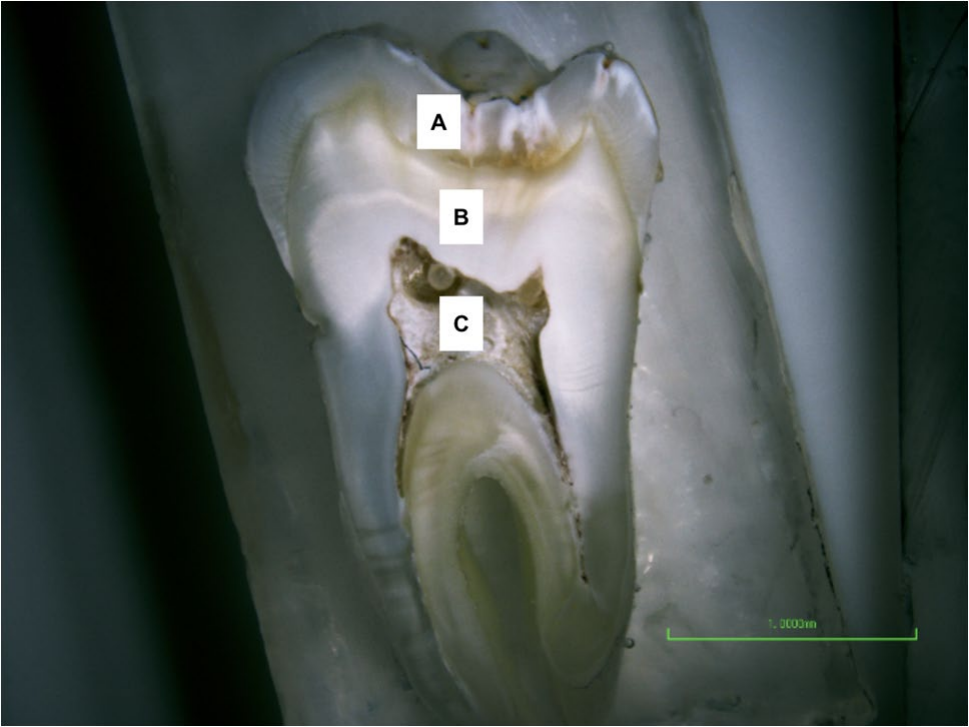
Different tissue layers present in a human tooth. **A** Enamel, **B** dentine, **C** pulp chamber

The thermophysical properties of the tooth is a factor in its thermal behaviour and depends on the microstructures of each tooth layer (Fig. 1). However, because the human tooth is a living tissue, the heat conduction process occurs in conjunction with physiological processes, including the fluid motion in the DTs and blood circulation in the pulp chamber. Dentinal fluid flow could improve the heat transfer within the pulp during temperature changes. The pulpal blood flow also influences the thermoregulation of pulpal soft tissue. The increase of pulpal blood flow rate during extra heating from hot foods or rotary dental procedures (above 42°C) works as a heat sink, while during cooling, e.g. from the water jet spray of a handpiece, the blood flow would maintain the temperature as a heating source [7].

### Residual dentine

Dentine acts as a thermal barrier against harmful stimuli. The flow of heat through dentine is proportional to the TC of dentine and inversely proportional to the thickness of the residual dentine [34]. The key material properties for heat transfer in teeth; the TC and TD values are both low for dentine [21]. Residual dentine is a critical factor in reducing heat transfer to the pulp with its thickness seeming to be the most important factor in determining pulpal protection. A thicker residual dentin layer results in a greater insulating effect, affecting the quantum of heat transfer to the pulp chamber during dental procedures [7, 12, 13, 21, 34, 35]. Thus, factors such as the type of tooth preparation (full veneer preparation on molars, three quarter preparation on molars or premolars) should be carefully considered this ultimately determines the amount of residual dentine and therefore the level of potential risk to the pulp arising from intrapulpal temperature rise [21, 34]. However, in the clinical situation, the thickness of prepared dentine is difficult to assess and therefore cannot be used to exclude thermal damages to the pulp [21, 36].

### Dentinal tubules

Factors such as the presence of dentinal tubules strongly impact the porosity, density and TC of dentine [7]. Dentinal tubules are a network of channels radiating outwards from the pulp cavity to the DEJ [1, 6, 7]. Thermal conductivity of dentine will vary with dentinal tubule density, orientation and structure (normal, transparent and reparative dentine, with reparative dentine being the formation of a tissue barrier by odontoblast-like cells following pulpal insults) [36]. For instance, the TC of dentine decreases with increasing volume fraction of dentine tubules [7]. Likewise, specific heat of dentine is said to rely on the orientation of dentine tubules [7]. These characteristics in dentine promote a better transfer of heat towards the pulp where heat dissipating mechanisms can be activated [7, 14]. Yet, these physical properties of teeth differ extensively even for a single tooth but also between different teeth (incisor, canine, molar) including age, gender, ethnicity and different donors [37–39].

Previous work has demonstrated that there is a notable increase in the number of dentinal tubules in regions near the pulp chamber, providing a greater overall surface area available for diffusion compared to a much smaller presence of dentinal tubules in regions closer to the DEJ [8]. This spatial variation in density of the dentinal tubules range from about 10,000 lumens/mm^2^ at the DEJ to about 60,000 lumens/mm^2^ near the pulp [6]. Therefore, it could be concluded that the microstructure of human dentine is adapting to not only withstand thermal alterations but also to dissipate heat towards the pulp chamber. Accordingly, it is postulated that the thickness of the residual dentine layer could determine the density of dentinal tubules, where small amounts of residual dentine thickness would be more prone to intrapulpal temperature increase due to a greater presence of dentinal tubules [8].

### Dentine thermal conductivity

By combining the residual dentine thickness with the coefficient of thermal conductivity of dentine, it is possible to establish the rate of heat flow from a thermal exposure at the surface of the cut dentine layer and establish the potential risk to the pulp tissue. This relationship is represented by a modified thermodynamic equation [35]:

$$H=\frac{KA\left(t_2-t_1\right)}D$$

*H* is heat flow through dentine per unit time, *K* is the thermal conductivity of dentine, *A* is the surface area exposed to the heat stimulus, *D* is the thickness of the residual dentine layer and *t*_2_ − *t*_1_ is the temperature difference.

This equation demonstrates that heat flow through dentine is directly proportional to the TC and inversely proportional to the residual dentine thickness [34, 35].

## Heat generated during tooth preparation

### Tooth preparation

**Fig. 2:**
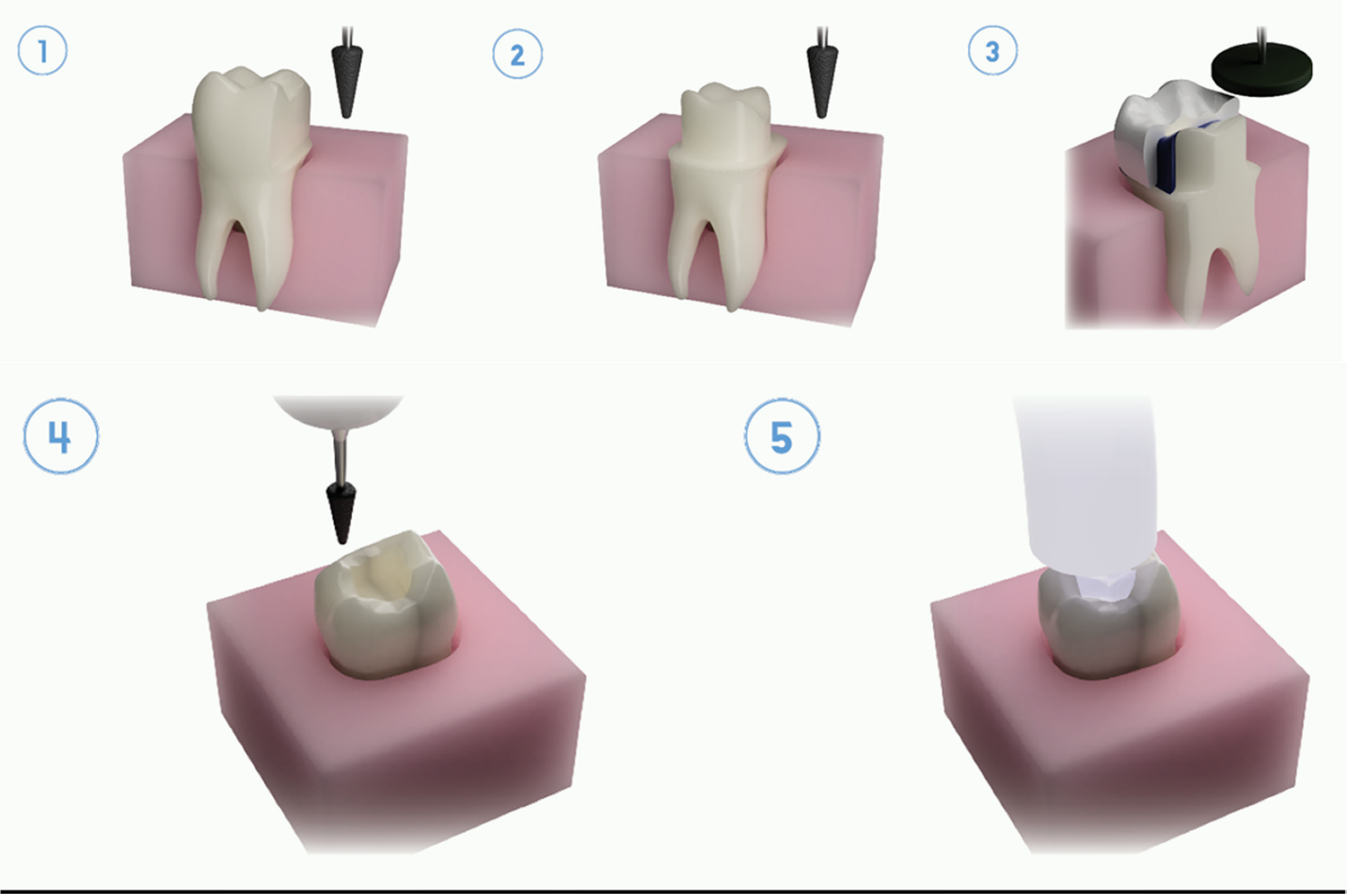
Different heat-generating procedures of high-speed dental handpieces. (1) Partial tooth preparation. (2) Full cuspal crown preparation. (3) Polishing procedure. (4) Bevelling of tooth preparation. (5) Light curing of dental material

The restorative process of preparing a tooth to receive a fixed prosthetic restoration requires both clinical and technical considerations [21] as shown in Fig. 2. A critical area of concern for the clinician during this, often lengthy and involved procedure, is the minimising of external factors that lead to an increase in heat production and are potentially harmful to the vitality of the tooth [13]. Two specific heat-generating variables include the friction between the HSDH and tooth and the exothermic setting reaction of self-polymerising restorative materials used for the temporalisation of the tooth preparation or the heat generated from the light curing of dental resins [7, 13]. Studies have shown a direct relationship between the tooth preparation design and intrapulpal temperature rise, especially the thickness of the residual dentine layer [13, 21, 34–36].

### High-speed handpiece in dentistry

In dentistry, the HSDH is a commonly used equipment in any clinical setting. It is used for fast and efficient removal of tooth structure in restorations [40]. A good high-speed handpiece should be of an ergonomic size and weight, have a suitable head size, have adequate power and speed, have adequate illumination and have sufficient cooling features. Cooling features are important because tooth cutting produces friction and heat between the bur and tooth surface. Excessive heat can transfer to the pulp, resulting in inflammation and necrosis if not dissipated efficiently, as well as structural changes in the enamel and dentine [26, 41].

### Air turbine versus electrical high-speed handpieces

There are two main types of HSDHs—an electric micromotor which utilises an electric motor to generate the required rotational force, and an air turbine which utilises compressed air [42]. The main advantage of electric micromotor–driven handpieces over air turbine handpieces is the greater cutting efficiency, with a smoother and even cutting rate caused by the constant torque maintenance under high loads and lack of ‘stalling’ compared to air motor–driven handpieces [43–45]. While air turbine far outruns electric motor run HDSH regarding speed, reaching speeds as high as 420,000 rpm, they lack the torque stability of electric HSDHs. Low torque means that there is less rotational force, and with the rotational speed decreasing it may stall at high loads, whereas the consistently high torque will maintain a constant rotational speed that does not decrease with high loads, therefore exhibiting a greater cutting efficiency [42, 46]. The greater cutting efficiency of electric HSDHs applies to a variety of dental materials, including glass ceramic, silver amalgam and high noble alloy [43]. The torque of the handpiece is expressed by the power specification of the handpiece [46].

One study found electric handpieces resulted in greater decrease in intra-pulpal temperature in comparison to air turbine handpieces, due to the improved cutting efficiency and friction production [42]. However, with no other studies validating this result, the impact of handpiece type on intrapulpal temperatures changes cannot be concluded. In addition, evidence of the effect of input air pressure and torque on temperature increase is conflicting between studies. Ozturk et al. [47] found temperature increase with increasing air pressure, but Firoozmand et al. [24] found no difference in pulpal temperature between high torque and low torque HSDH. Both speed and power of the HSDH is related to the generated energy, so increased HSDH speed results in increased intra-pulpal temperature [48]. However, within the handpiece body itself, more heat is generated with electric HSDHs. This can result in soft tissue injuries when the handpiece is running at maximum speed without an effective cooling mechanism [49, 50].

### Coolant ports to reduce the thermal shock to the tooth

Most modern HSDHs incorporate air or air-water coolant ports. These are designed to form a halo around the bur and spray high-velocity water and/or air at the tooth-bur interface. This improves visibility, cutting, polishing and cooling efficiency, as well as decreases the frictional heat and risk of pulp injury [23, 30, 40, 47, 51, 52]. Schuchard [53] conducted a photographic study looking at the action of the coolant water droplets while the bur is rotating at working speed. He found that with the water volume and pressure used in clinical setting, the coolant does not actually reach the cutting part of the bur. Instead, the coolant has a cooling effect on the entire tooth, rather than just the area of contact [53].

Dental handpieces can differ on the number and location of their coolant ports, with 1-, 3- and 4-port varieties available for air turbine, and 1- and 4-port varieties for electric. Siegel and von Fraunhofer [54] reported that the introduction of the 3- and 4-port handpieces was to allow sufficient cooling of the tooth if one or more ports become blocked. Theoretically, it would be hypothesised that the greater number of coolant ports would result in a greater cooling efficiency as the coolant would be greater distributed over the cutting surfaces. In general, manufacturers have claimed that more ports enhance cooling efficiency; however, the results from recent studies are inconclusive. Chua et al. [23] demonstrated that there was no statistical significance between the intra-pulpal temperatures following the use of high-speed air turbine handpieces with different coolant port designs (1-, 3- and 4-ports), whereas a later study, Lau et al. [55], found a statistically significant difference found between the cooling efficiency with 1- and 4-port coolant design on electric micromotor HSDHs.

The coolant port design also influences the cutting efficiency—HSDHs with a multiport coolant design exhibit greater cutting efficiency compared to those with a single coolant port, even if the 1-port handpiece has a higher coolant flow rate [54, 56]. Similarly, Lloyd et al. [57] observed that cutting with water results in cutting rates three times that of dry cutting, with Siegel and von Fraunhofer finding that 1-port HSDH had significantly lower cutting rates than 3- or 4-spray ports when making groove cuts (with intact edges) [54, 57]. However, this difference was only observed when performing groove cuts (surrounded by tooth structure), and not when performing edge cuts. Groove cuts differ as they produce greater increases in temperature, due to the concentration of generated heat at the bur interface and decreased accessibility of water spray [58]. Additionally, the position of spray ports affects water supply to cutting interface and therefore cutting rate. The authors Siegel and von Fraunhofer [54] observed in previous studies that cutting rates varied with different spray port numbers and positioning, especially if there was a blocked port. Yang and Sun [56] conducted a similar experiment on ceramic blocks, utilising both edge and groove cutting. However, they found that only the output coolant flow rate, and not the number of spray ports, affected cutting efficiency.

### Type of bur used

There are a variety of burs used by the dentists with HSDHs. Studies have concluded that the type of bur used, whether it be made of diamond or carbide, and/or its shape, size and grit size, depended on clinician preference and is highly influenced by equipment used during dental school [59]. Diamond burs are the most popular, followed by tungsten carbide burs [59].

Current studies on the effect of bur type on the heat generation and cutting efficiency are inconclusive, with studies reporting contradicting findings on the differences between diamond and carbide burs. Most agree that carbide burs generate less heat and pressure [60] potentially due to the different cutting mechanisms—diamond burs tend to clog with a grinding action used to remove tooth structure [61] whereas carbide burs, with their fluted design, use a cleaving action instead. Diamond burs have been found to showcase poorer cutting efficiency when compared to carbide burs with a thicker smear layer and greater frictional heat produced [42, 48, 62]. This can be attributed to the action of a diamond bur, where a large amount of energy is applied on the small cutting surfaces of each diamond grit [58]. Watson et al. [48] found that diamond burs produced more temperature increases, as there is a greater area of contact and more friction produced. Nevertheless, several studies have found the opposite with lower heat generation with diamond burs [61], and greater increases in temperatures with deeper cavity preparations using tungsten carbide burs [62].

Numerous other factors such as the bur size, shape, coarseness and amount of surface wear can influence the amount of heat generated during mechanical tooth preparation. Diamond burs are available in different grit sizes, which produce different finishes. Coarser grit burs produce less of a smooth surface, and more friction and thus heat [63, 64]. In addition, as burs wear out and lose their grit, there is reduced cutting efficiency [65, 66]. Similarly, when the diamond bur clogs with debris or stall, their generated energy creates a significant spike in temperature [58]. In addition, there appears to be differences in the burs produced within a manufacturer and between different manufacturers [61].

Overall, however, studies found that the increase in intra-pulpal temperature was not clinically significant. In Watson et al.’s study [48], all tests with different burs resulted in a drop in intra-pulpal temperature. Ercoli et al. [51] and Lau et al. [55] also found that despite an increase in temperature for some burs, all were still below the critical value to cause pulpal damage.

### Cutting technique

The cutting technique adopted by dentists can vary by either continuously cutting with no pause or intermittent cutting with periods of pauses. A study on cutting techniques showed that intermittent cutting produces greater cutting effectiveness [67]. In intermittent cutting, heat dissipation can occur during the periods of rest where the bur is not in contact, resulting in a lower overall temperature increase [64, 68, 69]. Similarly, an experimental study also showed that continuous cutting with high loads resulted in greater temperature increases [68].

Other variables of cutting technique, such as rotational speed, operator pressure, depth of the cavity preparation, duration and even the differences in the cutting medium (extracted teeth versus glass slabs), will also impact the heat generated and the results of previous in vitro studies.

### Effect of water coolant on intra-pulpal temperature

Many studies found that air coolant is insufficient, and water stream or air-water spray are more appropriate forms of cooling when drilling with HSDHs. This is supported by both histological studies [70, 71] and studies measuring intra-pulpal temperature [47, 48, 71–76].

The two main variables that influence the heat absorption ability of the water coolant are its flow rate and the temperature [27]. Currently, utilisation of flow rates between 30 and 50mL/min is the standard cooling conditions [27, 47, 51, 52, 68, 77, 78], with the International Organization for Standardization recommending the upper level of the threshold at 50mL/min [79]. These coolant flow rates are necessary to decrease thermal injury with a cooling effect at both the cutting interface and the handpiece head, with maintenance of adequate operator visibility [25, 80]. If the coolant flow rate is high enough, theoretically, the pulp tissues will not exceed the temperature of the water coolant used [41]. When sufficient coolant was used, the closer the bur is to the pulp, the further the temperature dropped [72]. Leung et al. [81] also found that thermal resistivity for air-water spray was lower than for water stream cooling at the same flow rate. The maximum output flow rate of the HSDH varies with the number of spray ports, if the water pressure is kept the same [80]. The study found that HSDH with 1 spray port had the highest flow rate, followed by 2 ports, and then 3 spray ports.

Temperature of the coolant water also affects intra-pulpal temp in cooling. Lower water temperatures have both a greater cooling efficiency and a greater heat absorption capacity [41]. However, the clinical usefulness is limited due to the increased risk of pulp damage with the reduced pulp blood flow and decreased waste removal ability. This can occur if pulp temperature drops below 21°C [82]. In addition, both the operator and patient may be uncomfortable when cooler water is used, especially with prolonged appointments and patients who suffer from cold sensitivity. Several studies have recommended the use of room temperature water at between 25 and 50 mL/min coolant flow rates to effectively prevent pulp injury [47, 52]. Farah et al. [41] investigated the impact of three different water coolant temperatures, 10°C, 23°C and 35°C, at the coolant flow rate of 50 mL/min. This study concluded that water coolant was essential to prevent injury of the pulp and soft tissues, and that a coolant temperature of 35°C in electric handpieces offers only minimal protection as temperature increases were observed. Past studies have shown that electric handpieces increase the water coolant temperature, with heat gained when the water travels through the handpiece [41], which results in the possibility of soft tissue damage [50]. Friction can be generated by the motor bearings, which can create heat that results in warming of the coolant water [68].

## Intrapulpal temperature increase by dental provisional crowns

After tooth preparation, the patient receives provisional crown(s) while the final dental restoration is made in the dental laboratory. The requirement for provisional crowns has been mainly derived from the methodological process that relies on the indirect fabrication of the definitive restoration in the dental laboratory. Provisional crowns must, with the exception to the type of material from which they are fabricated, resemble the planned final restoration in all regards to satisfy areas of critical concern [83]. These restorations restore function to the prepared tooth with only slight differences to the definitive restoration they precede [84].

The overarching aims of provisional crowns can be summarised as biologic, diagnostic, aesthetic and mechanical [85]. Biologically, provisional crowns must provide adequate contour that stabilises and promotes gingival health, and restore functional intercuspal and proximal contacts that prevent migration of the prepared tooth and movement of the adjacent teeth, as well as provide immediate protection to the pulpal tissues [86, 87]. The latter is of particular interest to this review as an unfavourable combination of the type of material and the method of fabrication could be detrimental to the pulp of a vital tooth. Therefore, clinicians must take extreme caution during the provisional restorative phase to ensure the health of the underlying tissues.

### Fabrication methods and types of dental provisional crowns

There are two main methods for fabricating provisional crowns: the direct and the indirect method. The direct method places acrylic resin material onto the prepared tooth, with the risk of thermal injury at temperature increases of 5.6°C in the pulp [18, 36]. Most chair-side materials in use by clinicians for provisional restorations lead to a rise in temperature during polymerisation [88], and may also cause irreversible damage to the gingival and pulpal tissue [89]. Furthermore, the presence of free monomer in direct contact with open dentinal tubules can be harmful and cause pulp inflammation if it leaches towards the pulp tissue [90, 91]. For the indirect method, materials are able to be cured in a hydro flask which shields the freshly prepared tooth from the heat released from the polymerising resin [83].

Polymers used in provisional restorative materials are classified either by their chemistry or by method of curing. The chemistry group of polymers include acrylics, composite resin and polycarbonate [92]. Methods of curing include chemical, heat, light or dual-activated. Predominately available commercial options for provisional restorations are either composite resin (bis-A-glycidyl methacrylate, bis-acryl, urethane dimethacrylate) or methacrylate resin (methyl methacrylate, ethyl methacrylate, vinyl methcrylate, butylmethacrylate)–based materials. The choice of material should be based on the clinical needs and longevity of the provisional restoration.

### Exothermic properties of provisional materials

Provisional crown materials available today have in common that they cure by radical polymerisation resulting in either a non (mono-methacrylates) or highly cross-linked polymer network (di- or multifunctional methacrylates) [36]. It is the exothermic character of radical curing that leads to a significant amount of heat being generated during the course of polymerisation [36]. The reaction of these polymer-based provisional materials is through additional polymerisation, where carbon-carbon double bonds are converted to new carbon single bonds. The exothermic heat released during the polymerisation process is a direct result from the difference in energy between the two bonds [21, 34]. Thus, there is variety in the amount of heat generated for different materials. For example, in a previous study examining the temperature profiles of a direct (Luxatemp) and preformed (Hi-tempo) provisional crown materials, both of which is placed directly on the tooth preparation, a higher temperature increase was noted with the preformed crown system [93].

## Heat generated during the placement of the final dental restoration

The placement of the final dental restoration often requires adjustments, with the generated frictional heat transferred to the pulp chamber [44]. This heat transfer will be dependent on the thermal conductivity and diffusivity of the material/materials being used in the construction of the final restoration and the material of the bonding system.

Generally, thermal conductivities increase in the following order: polymers < ceramics < metals [94]. The higher value of thermal conductivity means that the material has greater ability to transmit thermal energy. However, if the temperature gradient changes with time, thermal diffusivity is used to determine the amount of heat transferred. Therefore, the thermal diffusivity of a dental restorative material might be more important than its thermal conductivity. This property also depends on the material’s density and heat capacity. Thermal diffusivity is not in proportion to the thermal conductivity, which means that a material might have a low thermal diffusivity and relatively high thermal conductivity.

Gold is sometimes utilised as an alloy material for dental restorations which has about 500 times the thermal conductivity (297 Wm^−1^K^−1^) and 600 times the thermal diffusivity (1.18 cm^2^s^−1^) than that of dentine. Hence, compared to dentine, gold restorations provide very little protection to the pulp against the thermal stimulation. However, the thermal conductivity of zirconia (2.5–2.8 Wm^−1^K^−1^) is extremely low compared to metallic materials and alumina (30 Wm^−1^K^−1^) [94] with lithium disilicates having a thermal conductivity of 5.2 Wm^−1^K^−1^ [95].

### Intraoral polishing of fixed dental restoration

Temperature rise is a common occurrence and could easily exceed the 5.5°C threshold value during the intraoral polishing procedure [96]. Zirconia, for example, has much higher hardness, elastic modulus and fracture toughness than other all-ceramic restorative materials [97]. Therefore, it requires much higher frictional forces (e.g. with higher speed and/or harder polishers) to create a smooth surface if it is not glazed, which is known to generate more heat [97, 98]. İşeri et al. [99] studied the temperature changes during clinical procedures, focusing on the periodical and continual grinding of disc-shaped zirconia specimens (15mm diameter × 1mm) with micromotor at 22,000 rpm and high-speed handpiece at 320,000 rpm. The study showed that dry grinding and adjusting zirconia produced a temperature rise of 63.4°C, by far exceeding the critical temperature which is known to cause pulp damage.

Chavali et al. [98] investigated the influence of two polishing systems and three speeds on the heat production of zirconia. In order to compare the heat generation via intraoral polishing, three different types of polishing agents were used to polish zirconia specimens into 4-mm-thick sections at either 5000, 15,000 or 40,000 rpm with slow-speed dental handpieces [98]. The results showed that no group generated surface temperature over 42°C, which is just under the critical temperature for pulp damage reported by Zach and Cohen [5].

### Heat generation during direct restoration with light curing

The heat generated during photopolymerisation using visible light-curing units has the potential of causing damage to pulp tissue. The temperature elevation occurs due to increased exposure time to light during irradiation. Studies have identified photopolymerisation as a big risk to pulp health demonstrating a temperature rise between 4.3 and 7.5°C during photopolymerisation of composite discs [100]. Another study recorded intrapulpal temperature rises ranging from 1.5°C to more than 4°C during light-curing of composite resin restoration of extracted teeth. Yet, clinical experiments have demonstrated that the pulp appears to recover from transient heating from light-curing units [14]. Some consideration must be given to the combination of the temporary material and the type of light-curing unit used as its output may influence the final temperature rise. The heat emission during polymerisation may induce a temperature rise that may be of biological concern. With regard to tooth preparation in prosthodontic dentistry, the probability of damage to the pulp is real when the temperature increase due to polymerisation is greater than the physiologic heat dissipation mechanisms of the dental periodontal system [21].

### Influence of light intensity on temperature raise of BCRs

As discussed previously, the increase in light intensity is associated with increasing concern of heat generation within the BCRs and subsequent pulpal injury. Balestrino et al. [101] found a difference in heat generation between various types of light curing units. They concluded that the LEDs produced higher temperature rises than the QTH, and the LED with lower irradiance causing higher temperature rises than the LED with higher irradiance [101]. However, the heat dissipation design of a light-curing unit should also be taken into account. Armellin et al. [102] provided an alternative perspective on the heat generation of BCRs, by stating that the temperature increase during resin curing is a function of the rate of polymerisation, which is not only associated with the energy from light curing units, but also due to the exothermic polymerisation reaction and time of exposure.

Par et al. [103] found that temperature rise during curing ranged from 4.4 to 9.3°C and was significantly reduced by curing with the lower intensity blue curing unit. This study also suggested that the correlating temperature rise of radiant energy, in combination of material × thickness × curing unit, revealed a highly significant linear relationship [103]. However, there is no direct evidence that support the relationship between the light intensity on heat generation of BCRs and the subsequent pulpal injury. Uhl et al. [104] argued that no considerable difference in the temperature increase within the pulp chamber model was found for the different light curing units and composites.

## Methods to measure the change of pulpal temperature during dental procedures

The most used method for measuring heat generation is by measuring real time temperature change via thermocouples, which is a reliable and relatively simple method to measure temperature change within dental materials or measuring heat transfer across the tooth structure [5, 68, 74, 75, 105–115]. There have been variations in the methods of measuring heat generation which could potentially lead to differences in results. For example, the type of thermocouple wires used were different. Some studies used J-type thermocouples [101, 102] while others used K [104] or T-type [103]. However, there has been no evidence that suggest the types of thermocouples can have significant influence on the measurement of real-time temperature change in dental settings or dental materials.

Measurements of heat generation and related temperature change can also be affected by the position of the thermocouples placed, as a change in location of the probe, which can result in variation of measurements and inconsistent results [93]. Additionally, a silicone heat-transfer compound injected into the pulp chamber is used to help transfer the heat from the walls of the pulp chamber to the thermocouple [23, 93]. Measurements of heat transfer can be affected by the placement of the thermocouple, which needs to be in the same position at each measurement to minimise any variations that can be caused by any variant location of the probe [88]. Radiovisiography can be used to determine proper positioning of the thermocouple probe, as well as the residual dentine thickness [93]. To minimise this variation, radiovisiography can be adapted to aid in proper positioning of the thermocouple probe [102, 116].

Often specimens are prepared either in disc shapes or by using actual tooth preparations. As shown in Table 1, previous studies appear to largely employ molars for their studies with few using pre-molars and only two studies used dentine discs. In general, when using tooth specimens, the design had been shaped to represent real case applications by using tooth preparations for either crown or cavity restorations. From the literature, there also seems to be a systematic preference to using thermocouples to measure changes in temperature. Additionally, most studies have adopted some form of metallic material to fill the cavity of the pulp chamber to facilitate heat transfer to the thermocouple. On the other hand, it is surprising that most studies had a small sample size (*n*=5) and although two studies used water baths to simulate intraoral conditions, with only one study having attempted a model to simulate the complex intrapulpal fluid flow. Many studies listed in Tables 1, 2, 3, 4 had one thermocouple located in the pulp chamber. Thus, they were interested in only measuring the intrapulpal temperature change, rather than the heat transfer from outside the enamel down to the pulp (during dental procedures/restorative materials). The difference in methods adapted in various studies makes it difficult to compare their results. Furthermore, one common limitation of these in vitro studies is the lack of blood circulation seen in vital pulp and associated heat dissipation. Overestimation of the pulp temperature changes in in vitro studies is probable, with the lack of blood and dentine fluid flow, and lack of periodontal tissues [18–20]. This could limit the representativeness of the results to vital human dentition.

**Table 1:** Previous studies that investigated the changes in intrapulpal temperature caused by the exothermic setting reaction of resinous materials

Author	Title	Experimental design	Conclusion
Michalakis et al. [15]	Comparison of temperature increase in the pulp chamber during the polymerization of materials used for the direct fabrication of provisional restorations.	Mandibular molars with complete coverage restoration (1.5 mm axial, 2.0 mm occlusal reductions; 360° chamfer finish line); thermal probe inserted.	All materials resulted in an increase in pulp temperature; greatest with polymethyl methacrylate and no significant difference between polyethyl methacrylate, polyvinylethyl methacrylate and bis-acrylic.
Tjan et al. [35]	Temperature rise in the pulp chamber during fabrication of provisional crowns	Mandibular molars with complete crown restoration; silicone heat-transfer compound; thermocouple.	Temperature increases during fabrication of provisional crowns may be sufficient to damage pulp. Significant temperature reduction with use of silicone putty impressions.
Kim and Watts [34]	Exotherm behavior of the polymer-based provisional crown and fixed partial denture materials	A model test-cavity with polypropylene discs; type K thermocouple.	All materials exhibited an increase in temperature; Temphase with a significantly greater increase; no significant difference between Trim, Protemp 3 Garant and Luxatemp.
Castelnuovo and Tjan [12]	Temperature rise in pulpal chamber during fabrication of provisional resinous crowns	Mandibular molars; metal-ceramic restoration with 1.5 mm shoulder; heat transfer compound and thermocouple.	Temperature increases were observed for all resins; higher increase in resins with a polypropylene matrix.
Seelbach et al. [36]	Temperature rise on dentin caused by temporary crown and fixed partial denture materials: Influencing factors	Human dentine discs, coronal dentine specimen; NiCr-Ni-thermocouple.	Temperature increases may damage the pulp with material thickness ≥4mm, or remaining dentine ≤1mm.
Singh et al. [21]	Intrapulpal thermal changes during direct provisionalization using various autopolymerizing resins: *Ex-vivo* study	Premolars and mandibular molars, conventional ¾ crown and conventional complete veneer metal crowns	Both the type of provisional restoration material and preparation design impacts temperature increase; temperature increase polymethylmethacrylate > polyethyl methacrylate > Bis-acrylate composite; full veneer on molar > 3/4 molar > ¾ premolar preparations.
Daronch et al. [13]	Effect of composite temperature on in vitro intrapulpal temperature rise	Maxillary premolar, large class V preparation on facial surface	Preheated composites increased pulp temperature more than room-temperature composites.
Hannig and Bott [14]	*In-vitro* pulp chamber temperature rise during composite resin polymerization with various light-curing sources	Mandibular molar, class II cavity, K-type thermocouple.	High energy output curing lights significantly increase pulp chamber temperature, compared to the conventional curing light.
Dias et al. [93]	Real-time pulp temperature change at different tooth sites during fabrication of temporary resin crowns.	Two temporary crown systems (convention direct and preformed thermoplastic) on incisor, premolars and molars; K-type thermocouple.	Direct crown fabrication system showed minimal temperature changes with a larger change observed in the preformed thermoplastic system.

**Table 2:** Previous studies that investigated the changes in intrapulpal temperature caused by the water flow and use of HSDHs

Author (year)	Title	Experimental design	Conclusion
Cavalcanti et al. [68]	High-speed cavity preparation techniques with different water flows	In vitro study, bovine teeth; low and high-load techniques; thermocouples	Change in temperature values without water coolant was always above 5.5°C. Low-load technique and use of water coolants are required.
Cavalcanti et al. [74]	Pulpal temperature increases with Er:YAG laser and high-speed handpieces	In vitro study, bovine lower incisors; class V preparations (2-mm depth); thermocouples	Water cooling was essential to avoid destructive temperatures when using both HSDH and laser instruments
Ercoli et al. [51]	In vitro comparison of the cutting efficiency and temperature production of 10 different rotary cutting instruments. Part I: Turbine	In vitro study, macor blocks; diamond and carbide instruments.	Regardless of rotary instrument, adequate water flow does not cause harmful temperature increases; 40ml/min of room temperature coolant was optimal.
Ercoli et al. [42]	In vitro comparison of the cutting efficiency and temperature production of ten different rotary cutting instruments. Part II: Electric handpiece and comparison with turbine	In vitro study, macor blocks; diamond and carbide instruments.	Reduction in temperature with electric handpieces with better cutting efficiency and a lower mean temperature than air turbine.
Firoozmand et al. [24]	Temperature rise in cavities prepared by high and low torque handpieces and Er:YAG laser	In vitro temperature study, bovine incisors; Class V preparations (1.5-mm depth); thermocouple	Lower temperature rise with laser compared to handpiece with no group reaching beyond the 5.5°C threshold
Funkenbusch et al. [131]	Designed experiment evaluation of key variables affecting the cutting performance of rotary instruments	Fractional factorial experiment with macor blocks	Control exerted by the dentist (simulated by applied force) was the single most important factor affecting cutting efficiency. Water flow and number of coolant ports were not statistically significant
Lauer et al. [27]	Effects of the temperature of cooling water during high-speed and ultrahigh-speed tooth preparation	In vitro temperature study, third molars; thermocouples with location confirmed by radiography..	Important factors of pulpal temperature include cooling water under 35°C, adequate remaining dentine thickness and intervals between cutting
Ottl and Lauer [64]	Temperature response in the pulpal chamber during ultrahigh-speed tooth preparation with diamond burs of different grit	In vitro temperature study, third molars; NiCrNi thermocouples; cylindrical fine, coarse and ultra coarse diamond burs.	Coarse diamond burs generated greater temperature increases. Coolant water less than 38°C and short intervals between rests are beneficial to prevent temperature increase
Ozturk et al. [47]	In vitro assessment of temperature change in the pulp chamber during cavity preparation	In vitro temperature study, premolars, thermocouple	Decreasing water coolant amount or increasing air pressure and load increases pulp temperature. High coolant water flow rate (40ml/min) prevented high temperature increase
Gross et al. [132]	*In vivo* temperature rise and acute inflammatory response in anesthetized human pulp tissue of premolars having Class V preparations after exposure to Polywave® LED light curing units	In vivo thermocouple study; anaesthetised pulp tissue of first premolars; class V preparations and exposure to light.	No difference observed with similar radiant exposure values; no noticeable histological changes in pulp tissues with 5.5°C or greater temperature increases
Siegel and von Fraunhofer [54]	The effect of handpiece spray patterns on cutting efficiency	In vitro study, ceramic blocks, 6-mm edge and groove cuts	Cutting rates varied by cut type and number of spray ports. Cutting rate for 1-port was significantly lower during groove cut, but there was no statistical significance for edge cuts.
Watson et al. [48]	High and low torque handpieces: cutting dynamics, enamel cracking and tooth temperature.	In vitro temperature study; central incisors, premolars and molars; slot cuts; thermocouples	High-torque handpiece was better able to cope with increased loading. No evidence of increased tooth cracking or heating with high-torque HSDH, indicating that these do not have any deleterious effects on the tooth.
Yang and Sun [56]	Effect of the spray pattern, water flow rate, and cutting position on the cutting efficiency of high-speed dental handpieces	In vitro study; ceramic blocks	Author’s recommend multiport handpieces to be used with water flow rates greater than 30 mL/min.

**Table 3:** Previous studies that investigated the changes in intrapulpal temperature caused by the light-curing of restorative materials

Author	Title	Experimental design	Conclusion
Uhl et al. [116]	Influence of heat from light curing units and dental composite polymerization on cells in vitro	In vitro experiment; pulp chamber model; assessment of temperature change and cytotoxicity of composites (MTT test).	Factors such as temperature, light and presence of unpolymerised composite have a potential effect on fibroblast cells.
Haenel et al. [133]	Effect of the irradiance distribution from light curing units on the local micro-hardness of the surface of dental resins	Composite specimens were irradiated with micro-hardness measured.	Hardness distribution followed distribution of irradiance.
Balestrino et al. [101]	Heat generated during light-curing of restorative composites: Effect of curing light, exotherm, and experiment substrate	Composite samples (*n*=5) were placed on a thermocouple tip inside three substrates. The composites were photo-activated using three curing light at 1mm distance.	Temperature increase during curing of composites depends on curing light design, surrounding substrate (aluminium < Delrin and tooth) and composite type (methacrylate-based < silorane-based).
Armellin et al. [102]	LED curing lights and temperature changes in different tooth sites	In vitro thermal study; first molars; type-J thermocouples	Temperature increased during photocuring of composite; polymerisation reaction, light unit energy, remaining dentine thickness, radiation and duration of exposure were influencing factors.
Alkhudhairy [134]	The effect of curing intensity on mechanical properties of different bulk-fill composite resin	Cylindrical specimens of bulk-fill composites; Vickers microhardness, Compressive strength and Diametral tensile strength tests.	Higher curing light intensity (1200 mW/cm^2^) had a positive influence on mechanical properties for several specimens compared to lower intensity (650mW/cm^2^).
Daugherty et al. [135]	Effect of high-intensity curing lights on the polymerisation of bulk-filled composites	11 commercially available bulk-fill composites; depth-of-cure and degree-of-polymerisation measured after exposure times 3/9-s, 3/20-s, and 10/20-s.	Increasing exposure time increased depth-of-cure and degree-of-polymerisation; all composites did not meet the depth-of-cure advertised by manufacturers.
Shimokawa et al. [136]	Effect of light curing units on the polymerisation of bulk fill resin-based composites	Four light-curing units; two bulk-fill composites; Knoop Microhardness test.	Distribution of microhardness values varied between units, some more homogeneous than others. There was a positive correlation between radiant exposure and microhardness.
Sahadi et al. [137]	Multiple-peak and single-peak dental curing light on the wear resistance of bulk-fill composites	Two light-curing units; three resin composites (2mm thick, 10-mm diameter discs); investigated surface roughness, roughness profile, topography and microhardness after in vitro toothbrushing.	Increases in surface roughness and microhardness with reduction in composite volume was observed. No impact of curing unit on roughness profile.
Germscheid et al. [138]	Post-curing in dental resin-based composites	Six resin based composites (1mm thick, 9–10-mm diameter), five exposure times (20, 5, 3, 1.5 and 1 s) at radiant exitance of 1.1W/cm^2^.	Filler content determined the shrinkage rate; post-curing shrinkage was observed 15 h after exposure; shrinkage and degree of conversion related ageing rate increased with decreasing exposure time.
Par et al. [139]	The effects of extended curing time and radiant energy on microhardness and temperature rise of conventional and bulk-fill resin composites	Cylindrical composite specimens (8-mm diameter, 2 or 4mm thickness). Investigated the influence of material composition, curing unit type, and layer thickness on radiant energy, microhardness and temperature rise	Acceptable temperature increases and adequate curing efficiency was found for 30 s of curing. Curing time should be increased if concerned about undercuring of composites.
Marzouk et al. [140]	Effect of curing time on top to bottom microhardness of bulk-fill composite	Sixty cylindrical composite specimens (4mm × 4mm) were cured for 10, 20 or 40 s with an output of ≥ 1200 mW/cm^2^.	Increasing curing time increases the top to bottom microhardness of bulk-fill resin composites.
Ilie and Watts [141]	Outcomes of ultra-fast (3s) photo-cure in a RAFT-modified resin-composite	Output characteristics of the light curing unit were measured on a laboratory-grade spectrometer.	Comparable properties with RAFT polymerisation and conventional free radical polymerisation.

**Table 4:** Previous studies that involved experimental setup to stimulate intraoral environment

Author	Testing temperature	Humidity	Conditions in the pulp chamber	Method of temperature measurement
Chavali et al. [98]	Room temperature (24.0 ± 0.3°C)	Nil	Nil	Thermocouple inserted into cut-out (approximately 0.5mm from ceiling) of zirconia specimen and attached to a digital thermometer
İşeri et al. [99]	Room temperature	Nil	Nil	Temperature data logger mounted on the grinding apparatus with a magnet
Dias et al. [93]	Room temperature; water bath (37.5 ± 0.9°C)	Nil	Cleaned and filled with a thermal compound (Arctic Silver® 5, Arctic Silver Incorporated, Visalia, USA)	Thermocouple placed inside the pulp chamber and secured to the occlusal surface of the prepared tooth
Chua et al. [23]	Water bath (37 ± 0.6°C)	Nil	Cleaned and filled with a high-density polysynthetic sliver thermal compound (Arctic Silver 5; Arctic Silver Incorporated, USA)	Thermocouple inserted into the pulp chamber; position confirmed with radiographs
Park et al. [122]	Alcohol lamp (between 29 and 31°C)	Humidifier (relative humidity at 100%)	Nil	Thermos-hygrometer
Farah [123]	Incubator (37°C)	Nil	A curved half-circle needle inserted into the pulp chamber and attached to a peristaltic minipump with controlled fluid flow rate.	Thermocouple inserted into the pulp chamber; position confirmed with radiographs
Farah [41]	Incubator (37°C ± 1°C)	Nil	Cleaned	Thermocouple inserted into the pulp chamber; position confirmed with radiographs
Kodonas et al. [118]	Intrachamber temperature reached at 37°C	Nil	A needle inserted inside the pulp chamber and allowed circulation of 37°C distilled water at a 1 ml/min flow rate.	Thermocouples inserted into the pulp chamber and contacted the mesio-palatal and palatal surface of the tooth
Attrill et al. [127]	Room temperature	Nil	Cleaned and filled with a ‘pulp phantom’	Thermocouple inserted into the pulp chamber
Hannig and Bott [14]	Water bath (37 ± 0.1°C)	Nil	Cleaned and filled with water.	Thermocouple inserted into the pulp chamber; position confirmed with radiographs
Daronch et al. [13]	Thermostatically controlled water bath with intrapulpal temperature between 34 and 35°C	Nil	A plastic tube delivered water flow at 0.0125 ml/min and attached to one of the metal coupling tubes at the root end.	Thermocouple against axial wall of pulp chamber; position confirmed with radiographs
Walker et al. [130]	Environmental chamber (template internal conditions of 35°C)	Environmental chamber (template internal conditions of 90% relative humidity)	Nil	Nil
Bicalho et al. [129]	Electrical heater, sensor element and digital controls (37 ± 2°C)	Water spray system (preset relative humidity at 90 ± 4%)	Nil	Nil

### Experimental setup to simulate intraoral environment

Although thermogenesis during various dental procedures is extremely common, the amount of heat generation needs to be measured for providing clinical implications for better instruction and instrument application [36]. Heat transfer in teeth commonly depends on the geometry of the tooth itself, material properties and biological function. The biological function would be the biggest challenge for the experimental setup. In vivo experiments reflect the active processes within a tooth, whereas the experimental measurement of in vivo temperature changes within tooth pulp is impractical. Obviously, the in vitro test would be the only choice and the way how the simulation system is built up would influence the accurateness and reliability of the results. For mimicking the natural intraoral environment, three main factors should be considered: temperature, intra-pulpal blood fluid and humidity. Also, there are several integrated simulation systems of the intraoral environment used by previous studies, which could be referred to for setting up a more realistic and ideal experiment. Various methodologies applied across previous studies that stimulated the above three factors are summarised in Table 4.

### Effect of pulpal blood flow and microcirculation model

Pulpal blood flow (PBF) which varies with external stimuli helps maintain pulpal temperature by providing circulation and absorbing or providing heat [117]. Kodonas et al. [118] reconstructed pulpal microcirculation by running 37°C water through extracted human teeth and found significantly lower temperature increase under the microcirculation model. However, PBF varies with external stimuli. It decreases when the pulp is cool and increases significantly when pulpal temperature increases above 42°C and clinically used vasoconstrictors slow or stop PBF [119].

Most studies evaluating the heat generation via dental procedures, such as Chavali et al.’s study [98], were designed and completed at room temperature (24.0 ± 0.3°C) and with ambient humidity [34, 99]. However, the surface temperatures of the dentition and soft tissues have been found between 30 to 35°C and 32 to 37°C, respectively [120]. Amsler et al. [121] showed that the temperature range of the oral cavity was 26 to 29°C.

**Fig. 3:**
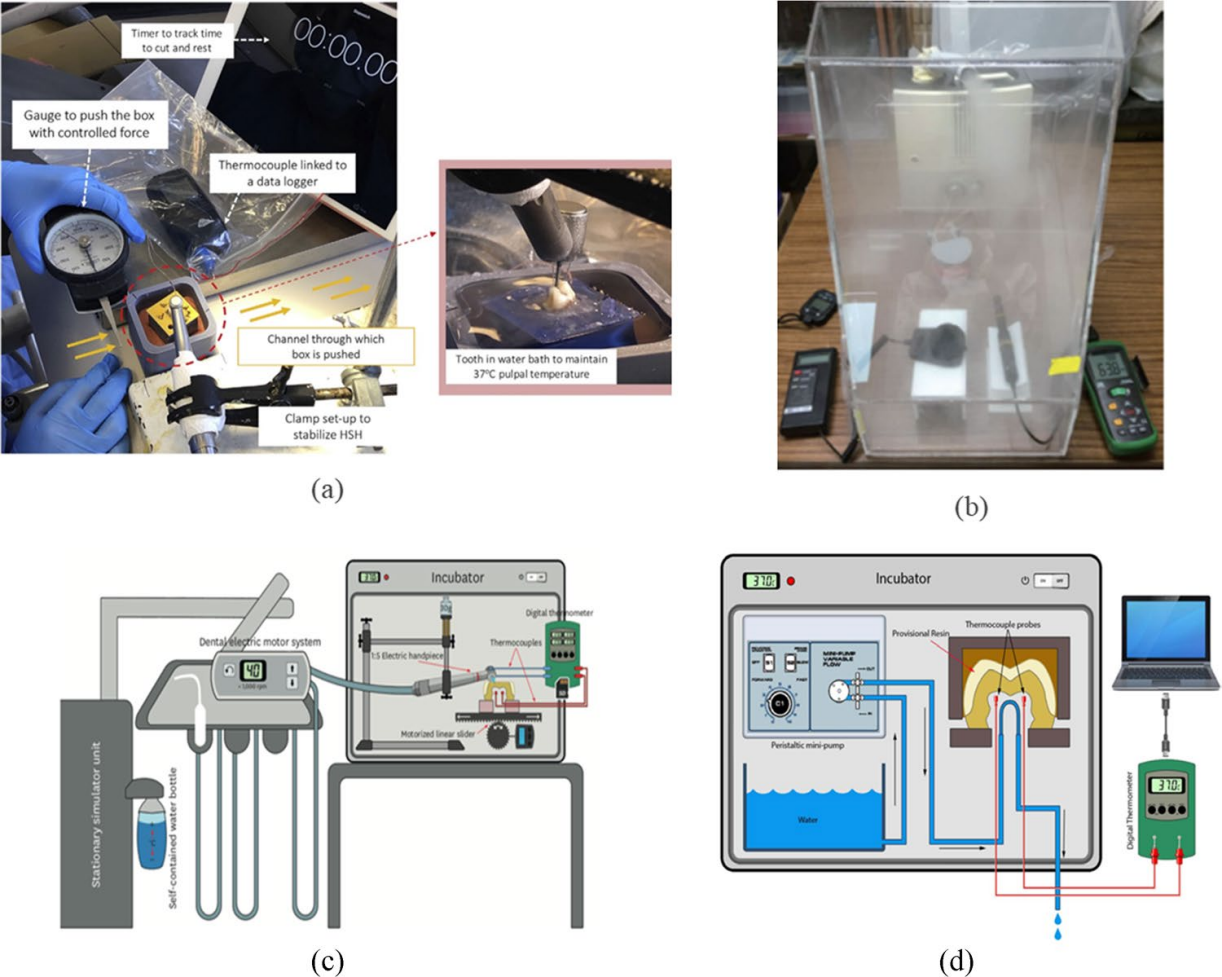
The example of intraoral simulation system; (**a**) Chua et al. [23]; (**b**) Park et al. [122]; (**c**) Farah [41]; and (**d**) Farah’s [123] experimental setup

**Fig. 4:**
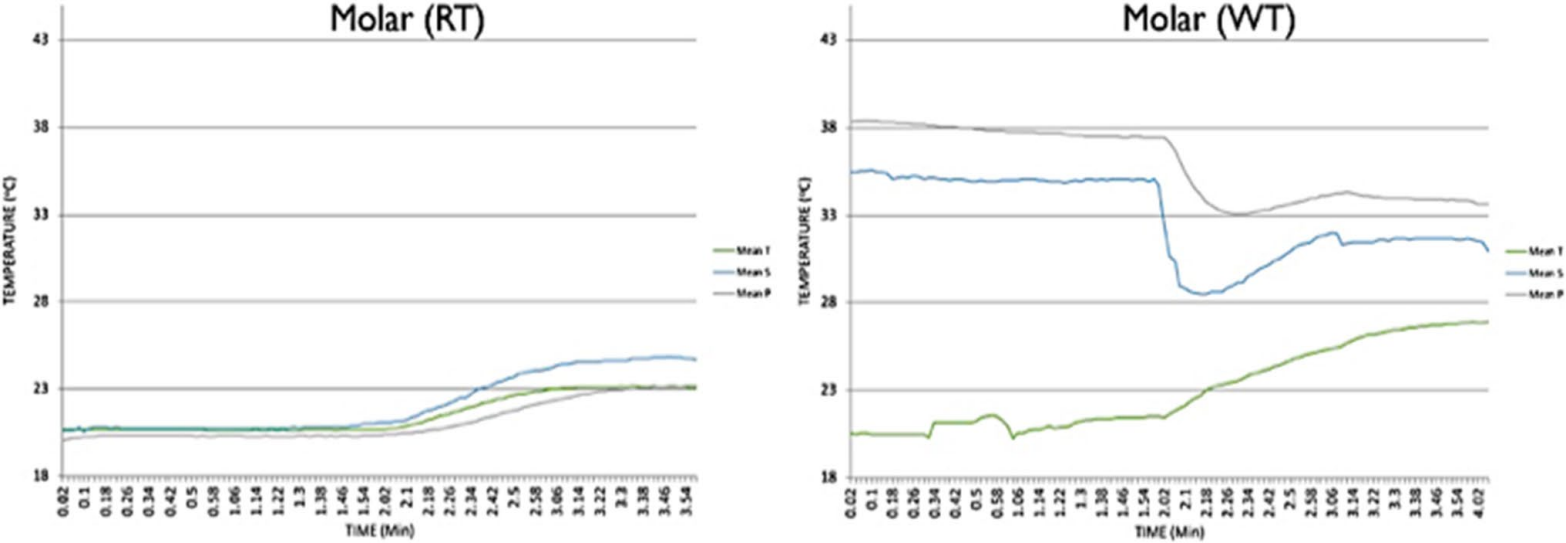
Different temperature profile graphs found from Dias et al.’s study [93] with (37°C water bath; WT) and without (room temperature; RT) pulp temperature simulation.

In Dias et al.’s study [84], they investigated the real-time pulp temperature change during temporary crown fabrication, comparing the heat generation during two different temporary crown systems and at different tooth sites. In Chua et al. and Dias et al.’s studies [23, 93], the authors simulated the pulp temperature by adding 37°C water in the container where the teeth specimens were fixed during the experiment (Fig. 3). These two studies highlighted the importance of conducting the experiment with 37°C water to simulate the baseline pulp temperature as the experiments carried out at room temperature had a significant impact on the temperature profile. For example, when pulp temperature was measured with and without 37°C water during a self-polymerising temporary crown fabrication, there was almost 20°C difference in pulp temperature between the two techniques (Fig. 4). When compared to the results from a previous study by Kim and Watts [34] using the same crown material conducted at room temperature, the authors found that while the pulp temperature stabilised at 37°C, the temperature recorded in the pulp chamber was 69 times lower [34, 93].

In Chavali et al.’s study [98], the polishing was also assessed without pulp temperature simulation. They discussed that dry polishing had the possibility to affect the rate of evaporation and thereby cooling rate. Their results of the temperature increased to 42°C from the intraoral polishing may have come from the fact that the experiment was conducted at room temperature and may have increased when it was done under pulp temperature simulation conditions [98]. In order to determine the reference values of the two intraoral factors, Park et al. [122] assessed the accuracy of two intraoral scanners utilising a box-shaped intraoral environment simulator to mimic the temperature and humidity of the mouth (Fig. 2b). Then, in Farah’s studies [41, 123], an incubator at 37°C ± 1°C was used as the simulation chamber of the intraoral temperature to evaluate the effect of cooling water temperature on the temperature changes in the pulp chamber (Fig. 2c, d). These two studies successfully simulated the intraoral temperature, but pulp flow and intraoral humidity were not simulated in this study.

### Intra-pulpal blood flow and intraoral humidity

Surveys such as that conducted by Goodis et al. [124] and Mülle and Raab [125] showed that the pulp blood flow probably mediated the effective homeostatic mechanism within human teeth, while many in vitro heat transfer studies of human teeth were carried out with cleaned and empty pulp chambers. Linsuwanont et al. [126] reported that, under temperature fluctuations, any fluid movement either away from or towards the pulp would inevitably result in the redistribution of the pulp chamber temperature. Lin et al. [7] also briefly stated that the TC and heat capacity of teeth of empty pulp chambers were significantly different from filled chamber with pulp soft tissue.

**Fig. 5:**
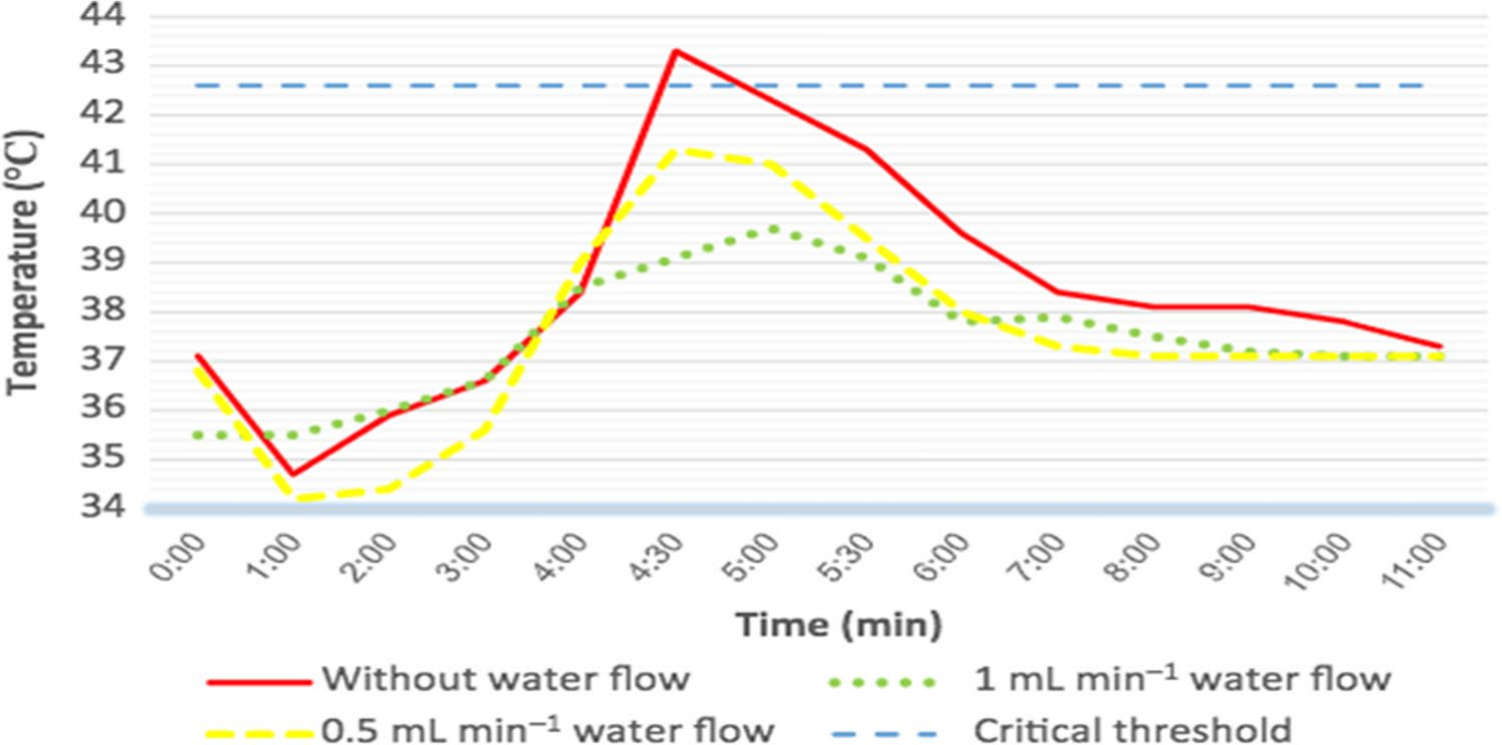
Temperature recorded in the pulp chamber during fabrication of temporary crowns; note the effect of different conditions of water flow rate [123]

In Kodonas et al.’s research [118], it found that the heat transfer experiments conducted without pulpal simulation would result in temperature increase of a greater magnitude than those with pulpal simulation. In order to simulate the vital dental pulp, Hannig and Bott [14] filled the pulp chamber with warm water to mimic heat transfer through soft tissue in the pulp chamber. Attrill et al. [127] filled the dead space of pulp chamber with a ‘pulp phantom’ which provided a thermal conduction environment similar to the vital dental pulp and Chua et al. [23] and Farah [41] utilised a high-density polysynthetic silver thermal compound inside the pulp cavity to improve conductivity. Nevertheless, Hannig and Bott [14] reported that the influence of pulpal blood flow on the thermal behaviour of the dentine-pulp complex cannot be simulated by stationary water inside the testing container. Chua et al. [23] also suggested that a better pulpal simulation experimental setup would help to find the more exact results of the temperature change. However, this will be challenging to accurately replicate due to its dynamic nature and changes in flow following different stimulations, such as temperature increases causing an increase in blood circulation [14]. Previous studies have many attempts to simulate this flow. An earlier research, Daronch et al. [13], noticed the deficiency of empty pulp chambers, which limited the direct application of the measurement data of in vivo situations, and employed an infusion pump connected to the tooth roots through a small diameter tube. This device delivered water at a speed of 0.0125 ml/min to simulate the pulpal blood flow. At the same time, the tooth was immersed into a water bath up to the cement-enamel junction. Then, Farah [123] used a curved needle connected to a peristaltic pump with a controlled fluid flow rate to simulate the pulp blood flow. This study also concluded that simulated pulpal blood flow resulted in a lower increase in the pulp chamber temperature, compared to when pulpal blood flow was simulated [123] (Fig. 5).

The relative humidity of the oral cavity has been found to vary in the range 78 to 94% during operative dental procedures [120, 121, 128]. Breathing through either nose or mouth showed no significant effect on the relative humidity [121]; however, the relative humidity would have decreased once the use of the rubber dam was completed [120]. Bicalho et al. [129] constructed a chamber to mimic the oral environment and evaluated the effect of the temperature and humidity. They controlled the humidity by a water spray system which was activated automatically to maintain a pre-set humidity value of either 50 or 90% at 22 or 37 °C. According to their study, the temperature and humidity had a significant influence on the mechanical properties of restored teeth with composite resins [129]. In another study, the flexural modulus and flexural strength properties of composite were not negatively influenced by the simulated intraoral conditions of 35°C at 90% relative humidity [130].

## Conclusion

Various steps of dental restorative procedures have the potential to generate considerable amounts of heat which can permanently damage the pulp, leading to pulp necrosis, discoloration of the tooth and eventually tooth loss. Thus, measures should be undertaken to limit pulp irritation and injury during procedures. This is especially true as damage to the pulp is accumulative and past insults affect the restorability of the tooth. Despite the importance of this topic, there are limited studies available which investigate the influencing factors and dental procedures. Experimental setups of simulating intraoral environment have been employed by most previous studies using an incubator at 37°C to mimic the intraoral temperature [23, 93, 123]. However, there is limited research which simulated the pulp blood flow using the peristaltic tubing pump and temperature [123]. The use of intraoral humidity chamber was employed by one study to simulate the relative humidity around natural teeth, which is an important variable [122]. This highlights the gap for future research and a need for an experimental setup which can simulate pulp blood flow, temperature, intraoral temperature and intraoral humidity to accurately simulate the intraoral conditions and record temperature changes during various dental procedures.

## Data Availability

Data will be available upon request.
